# Posttraumatic Growth Among Suicide-Loss Survivors: Protocol for an Updated Systematic Review and Meta-Analysis

**DOI:** 10.2196/64615

**Published:** 2025-02-14

**Authors:** Spence Whittaker, Susan Rasmussen, Nicola Cogan, Dwight Tse, Bethany Martin, Karl Andriessen, Victor Shiramizu, Karolina Krysinska, Yossi Levi-Belz

**Affiliations:** 1 Department of Psychological Sciences and Health University of Strathclyde Glasgow United Kingdom; 2 Centre for Mental Health and Community Wellbeing Melbourne School of Population and Global Health University of Melbourne Melbourne Australia; 3 Faculty of Social & Community Sciences Ruppin Academic Center Kfar Monash Israel

**Keywords:** posttraumatic growth, suicide-loss survivors, trauma, systematic review, meta-analysis, posttraumatic, suicidal, systematic review, meta-analysis protocol, traumatic impacts, bereaved survivor, social support, bereavement, data collection, sociodemographic, psychological, databases

## Abstract

**Background:**

Losing a loved one to suicide is an event that can have strong and potentially traumatic impacts on the lives of the bereaved survivors, especially regarding their grief, which can be complicated. These bereaved individuals are also less likely to receive social support following their bereavement. However, besides these adverse impacts, growing evidence supports the concept of posttraumatic growth following suicide bereavement. Posttraumatic growth is the personal improvement that occurs as a consequence of experiencing a traumatic or extremely challenging event or crisis. Only 1 systematic review and meta-analysis on posttraumatic growth following suicide bereavement has been conducted; this protocol is for the planned systematic review and meta-analysis update of the original systematic review and meta-analysis, as the original review collected its data in 2018.

**Objective:**

This review aims to investigate demographic characteristics, correlational relationships, and facilitative factors of posttraumatic growth in individuals bereaved by suicide. In addition, as this is an update of a previous systematic review and meta-analysis, we aim to compare our findings with the original review and to identify any similarities or differences.

**Methods:**

This protocol outlines the planned procedures of the updated systematic review and meta-analysis. MEDLINE, PsycINFO, Embase, CINAHL, Scopus, and Web of Science (Core Collection) were examined, and the search results were imported to Covidence, where title and abstract screenings and full-text screenings occurred. The inclusion and exclusion criteria for this updated review match those in the original review: (1) the study population must contain participants bereaved by suicide, (2) the study data must be quantitative, and (3) the study must report data on posttraumatic or stress-related growth. The original review conducted its search before 2019; thus, this updated review searched databases for the timeframe of January 2019 to January 2024. The updated meta-analysis will synthesize data from both the original and updated reviews to examine trends over time. The Newcastle-Ottawa Scale (NOS) will be used to assess publication quality. Random-effects meta-analyses will be conducted using RStudio (R Foundation for Statistical Computing).

**Results:**

The review was funded in October 2023 and is currently in progress. Results are expected to be finalized in October 2024. There are 21 articles that have been included in the review and are being analyzed at this time. We aim to submit the full article for publication in December 2024.

**Conclusions:**

The results of this updated systematic review and meta-analysis will be used to examine key relationships and findings regarding posttraumatic growth in individuals bereaved by suicide. The discussion will also investigate the findings of this updated review in comparison to the findings of the original review. Any differences would be highlighted. Limitations of the current review will be discussed, such as the quality of the articles included.

**Trial Registration:**

PROSPERO CRD42024485421; https://tinyurl.com/3hzpnzr3

**International Registered Report Identifier (IRRID):**

DERR1-10.2196/64615

## Introduction

When someone dies by suicide, those who lose this individual in their lives often face significant stress. These feelings can be inundating and are often accompanied by a sense of complicated grief and, at times, depression [1]. It is a tragic event that can generate negative emotions as well as many questions that may be left unanswered in the minds of these surviving individuals. In addition, this population of people bereaved by suicide is at an increased risk of suicidal behavior themselves [2]. Based on data that over 700,000 people die by suicide each year globally [3], and that for each suicide, there are from 6 family members to 135 community members considered to be bereaved or exposed, respectively [4,5], up to 94.5 million can be affected by suicide annually. Thus, many people affected by suicide loss each year are subsequently at increased risk of dying by suicide themselves.

There are commonalities between bereavement of suicide and other forms of death; however, some features of suicide bereavement are more pronounced, such as feelings of guilt, blame on self or others, or a longing for answers [6,7]. While all bereaved people may experience feelings of grief, loss, and depression, people bereaved by suicide specifically can begin to develop symptoms of posttraumatic stress disorder (PTSD) [8,9]. However, some individuals show signs of posttraumatic growth (PTG) more so than symptoms of PTSD. PTG is the personal improvement that occurs as a consequence of experiencing a traumatic or extremely challenging event or crisis. Literature has begun to show that, on average, PTG occurs more often than the development of pathological disorders following exposure to a traumatic event [10,11]. This is, of course, not to say that PTG is always the result of trauma, but rather, psychological suffering allows the opportunity for a person to grow and for new meaning to flourish in the face of trauma. An inverted U-shaped curve best describes the relationship between the developments of PTG—both too much and too little suffering are detrimental to the development of PTG [12,13]. This concept of PTG following a traumatic event [14] has since been applied to learning more about the bereavement experiences of individuals bereaved by suicide. Some authors have also used the phrase “personal growth” [15] or “stress-related growth” [16] in place of PTG. This study will use the terminology of PTG rather than personal growth or stress-related growth.

Determining a metric for PTG can be difficult as it is seen as both an ongoing process and a final result. However, the Posttraumatic Growth Inventory (PTGI) has been developed and shown to capture and highlight well many of the defining characteristics of PTG [10]. These characteristics are broken down into 5 domains: “greater appreciation of life and changed sense of priorities; warmer, more intimate relationships with others; a greater sense of personal strength; recognition of new possibilities or paths for one’s life; and spiritual development” [14]. It does not, however, fully account for all idiosyncratic differences that occur over time; in this regard, a more longitudinal approach for the measurement of such a complex construct (ie, PTG) could provide valuable insight [17].

For those who lose someone to suicide, there can be variables that affect their desire or willingness to seek both formal and informal help and work towards the development of PTG. Each of these variables can influence how well someone bereaved by suicide copes with and grows following their loss. For example, some individuals who have experienced suicide bereavement have reported that their primary support came from nonprofessional sources and that they were disappointed by their family and friends’ responses to their bereavement [18]. Suicide and the survivors’ grieving process can also be seen from a variety of perspectives depending on the culture from which someone comes, such as being seen as stigmatized, taboo, and isolating [19,20]. Along with these responses, some individuals have reported generally negative attitudes toward professional support systems, such as tactlessness and being unaligned in the grieving process [21]. Unfortunately, these detrimental experiences can be an additional stressor on top of what can already be a tragic and intense time of grieving. These factors could contribute to why up to 25% of people bereaved by suicide receive neither informal nor formal support [22]. Each of these variables (ie, poor support, stigmatization, tactless professional help, etc) can hinder PTG development.

This systematic review and meta-analysis is an update of a previous systematic review and meta-analysis conducted by Levi-Belz et al [23]. Our searches have found that the latter is the only one to ever be conducted on this topic. As their original review gathered data from literature before 2019, this review will include data found from searches between January 2019 and January 2024. This updated meta-analysis will synthesize data from both the original and updated reviews to search for any new or consistent trends. As some authors and databases call for a systematic review to be updated every 2 years [24], and with the paucity of understanding that exists on this subject, examining new literature, in conjunction with the previous review, could allow for a more in-depth understanding of which factors facilitate PTG in people bereaved by suicide. The aforementioned evidence suggests that PTG can and does occur following suicide bereavement; therefore, investigating further which factors might facilitate, as well as detract from, PTG development could greatly benefit people bereaved by suicide.

We have three primary aims with this review: (1) to investigate whether PTG can occur in the aftermath of a suicide loss, (2) to examine the sociodemographic and psychological correlates of PTG among people bereaved by suicide, and (3) to observe which factors facilitate PTG in the aftermath of suicide bereavement.

## Methods

### Overview

This systematic review will locate and summarize applicable data from the peer-reviewed literature [25]. The findings will be reported using PRISMA (Preferred Reporting Items for Systematic Reviews and Meta-Analyses) [26] formatting and follow the PRISMA-P (Preferred Reporting Items for Systematic Reviews and Meta-Analyses Protocols) checklist [27]. A meta-analysis will be conducted using the data extracted from this update as well as the data from the original review.

### Inclusion Criteria

#### Population

Study populations must include individuals bereaved by suicide. No limitations on age will be implemented.

#### Study Design

In accordance with the original review, only quantitative studies that report data on PTG will be included. While qualitative studies could offer a more comprehensive perspective on the topic, this update excluded them in order to follow the criteria of the original review. Gray literature and dissertations will be excluded such that peer-reviewed studies will be the only data involved. This was also done to follow the parameters set by the previous review.

#### Concept

Studies must report data on PTG in individuals bereaved by suicide. Studies that report data on PTG following various forms of bereavement but which do not separate the effects of suicide bereavement from other forms of bereavement will be excluded.

#### Context and Date of Publication

This systematic review and meta-analysis is an update of a previous systematic review and meta-analysis that gathered data before January 01, 2019. This update reviews literature published on or after January 01, 2019, and uses the same inclusion criteria as the first study. This updated meta-analysis will include the data from the 2019-2024 range as well as the initial study’s findings so as to paint a full picture.

#### Language and Location

There are no restrictions on language or location.

#### Search Strategy and String

As this review is an update of a previous systematic review, the inclusion criterion for the date range is publication on or after January 01, 2019. The initial systematic review, upon which this update is based, included all dates up to December 31, 2018, in its search. Databases searched include MEDLINE (through Ovid Platform), PsycINFO, Embase, CINAHL, Scopus, and Web of Science (Core Collection). We used the MeSH (Medical Subject Headings) terms “Posttraumatic Growth, Psychological,” and “Suicide” in databases that allowed for them (ie, MEDLINE [through Ovid Platform] and [PsycINFO])—the rest of the search string was free text and was used for each of the 6 databases mentioned. Multimedia Appendix 1 shows the search included in each database.

### Data Extraction

Using Covidence [28], a title and abstract screening was conducted by 2 reviewers (SW and BM) to exclude studies outside the criteria as well as to remove duplicate search results. A further full-text screening was performed in Covidence by the same 2 reviewers (SW and BM), and studies deemed inapplicable were excluded; reasons for exclusion of these studies were recorded. Any disagreements on the inclusion or exclusion of an article by the 2 reviewers were brought to the review team for further opinion to resolve the dispute. Data extracted included author’s name, year, location (country), study design, sample size, participants’ age and sex distribution, participants’ time since onset of bereavement, participants’ relationship to the deceased, outcome measures, names of the instruments used, and primary findings of the study. To allow for the analysis of subgroups, we also extracted information related to demographic factors, loss-related factors, intrapersonal factors, and interpersonal factors (see the *Analysis of Subgroups or Subsets* subsection). The authors of the original review shared their data from the first review; it will be used for the meta-analysis portion of this update.

### Risk of Bias (Quality) Assessment

The risk of bias will be assessed using 2 adapted versions of the Newcastle-Ottawa Scale (NOS) [29]. One adapted version will be for cross-sectional studies (7 questions), and the other for longitudinal studies (8 questions). This tool allows for the overall quality of a study to be assessed by the summation of “stars,” which each question can provide based on quality. Questions are broken into categories of “selection” (4 questions), “comparability” (1 question), and “outcome” (2 questions for cross-sectional and 3 questions for longitudinal). The highest grade a study can receive is 8 stars for cross-sectional studies and 9 stars for longitudinal studies. The total number of stars is then used to determine a quality ranking for each study, where quality levels range from poor (<5 stars), fair (5-6 stars), and good (6-8 stars) in cross-sectional studies and poor (<5 stars), fair (5-6 stars), and good (7-9 stars) in longitudinal studies. Studies from all quality levels will be included as there is a paucity of literature on this topic; however, the inclusion of any “poor quality” articles will be addressed in the limitations of this updated systematic review and meta-analysis.

### Strategy for Data Synthesis

The analysis will be conducted in RStudio (R Foundation for Statistical Computing) [30]. When studies did not report *r* coefficients, raw effects will be converted to *r* coefficients using the R package *effectsize* (version 0.8.3) [31]. Before conducting the analysis, we will apply a Fisher’s *r*-to-*z* transformation to the extracted effect sizes. Sampling variances and standard errors for the effect sizes will be calculated using the R package *esc* (version 0.5.1) [32].

Random-effects meta-analyses will be conducted using the R package *metafor* (version 4.4.0) [33]. This approach posits that individual study effects deviate not solely due to sampling error but also stem from another source of variance [34]. Heterogeneity will be assessed by Cochran Q, *I*² statistics, τ², and prediction intervals as recommended by Borenstein [35]. Publication bias will be assessed visually through a contour-enhanced funnel plot [36] and also by Egger regression test. To identify and assess the impact of potential outliers on the pooled effect and heterogeneity, influential analysis will be conducted using the R package *dmetar* (version 0.1.0) [37], using the leave-one-out method and the Baujat plot.

### Analysis of Subgroups or Subsets

In line with the original review, moderating factors will be categorized into 4 categories, with effect sizes calculated for each subsequent variable. The following examples are from the original review:

1. Demographic factors (eg, age, gender, race, religious affiliation, educational level, marital status, and voting or civil involvement).
2. Loss-related factors (eg, grief experience, time since loss, and closeness to and type of relationship with the deceased).
3. Intrapersonal factors (eg, resilience, coping, rumination, personality, optimism, and emotional experience).
4. Interpersonal factors (eg, self-disclosure, social support, help-seeking, suicide stigma and secrecy, interpersonal burdensomeness, lack of belonging, and attachment style).

## Results

**Figure 1:**
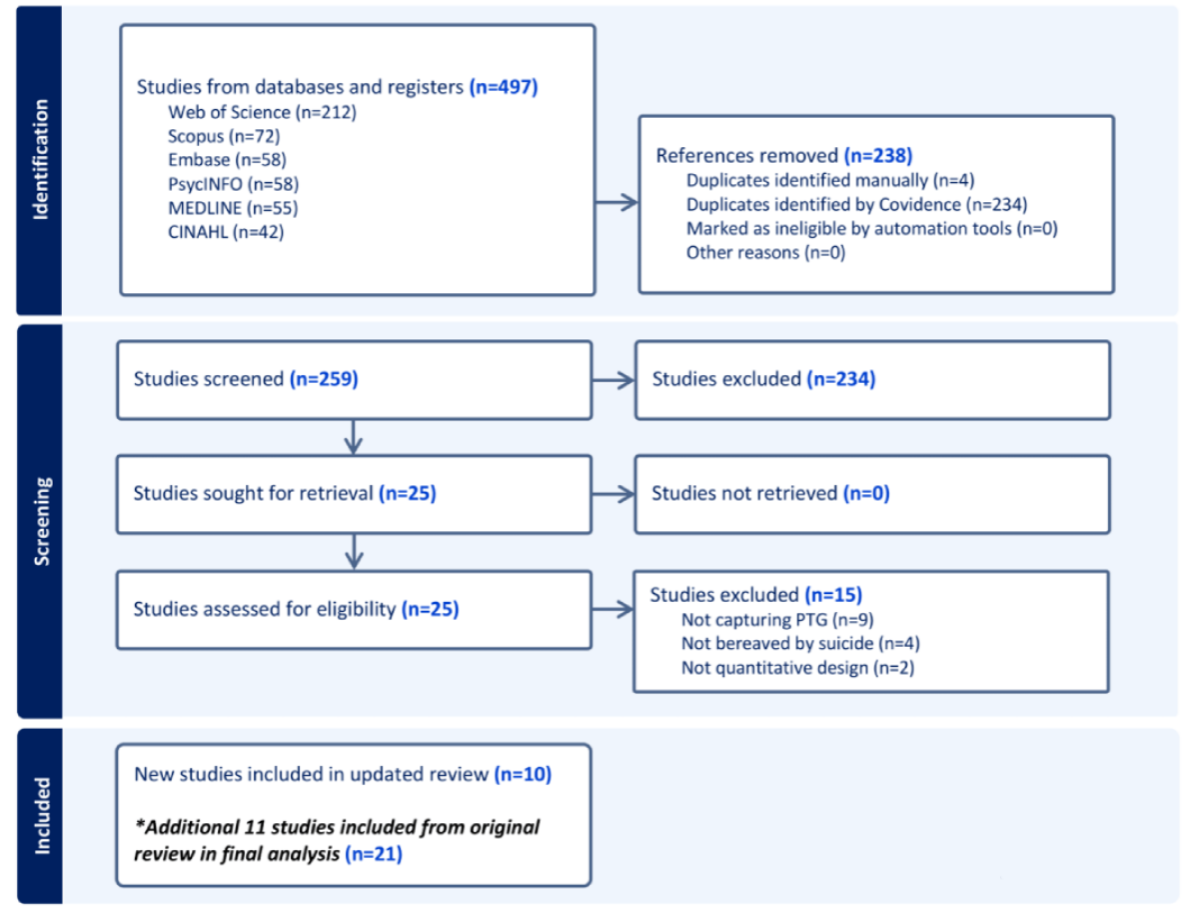
PRISMA (Preferred Reporting Items for Systematic Reviews and Meta-Analyses) flow diagram. PTG: posttraumatic growth.

This updated systematic review and meta-analysis was funded in October 2023 and is currently underway. It is expected to have results in October 2024. A total of 21 studies are included in this review; this will be reported by a PRISMA flow diagram (Figure 1). These 21 studies are currently being analyzed. All data produced in this review is included in Multimedia Appendix 2. A meta-analysis will be conducted using the data from both the studies of this updated search as well as the studies from the original review’s search. Doing so will allow for any varying or static trends to be revealed. Moderating factors will be examined to determine which variables may influence PTG development in people bereaved by suicide. We aim to submit the full paper for publication in December 2024. The PRISMA-P checklist will be abided by to ensure a higher quality of research practices are followed (see Multimedia Appendix 3).

## Discussion

We hypothesize that interpersonal variables of self-disclosure and social support, as well as the loss-related factor of time since loss and the intrapersonal variable of resilience, will have a positive influence on PTG. A full discussion will further examine these key relationships and general findings identified in the Results section. These findings will then be discussed in relation to 3 research aims identified in this review. The discussion will also investigate the findings of this updated review in comparison with the findings of the original review. Any differences would be highlighted and expounded upon to discover which factors have changed. Overall, we anticipate that the moderating variables will have varying effects on PTG and will deliberate on any potential correlational relationships, as this could emphasize which specific factors are correlated to greater PTG facilitation.

Implications and strengths, future work, and subsequent research, which could build upon the findings of this updated review will be discussed. The limitations of this review will be considered as well, such as the quality of the journal articles included in this review. Similarly, we will discuss how this systematic review has chosen to exclude qualitative articles as well as gray literature and dissertations and the strengths and limitations that follow this decision. We will also examine the impact of using solely the NOS for quality assessment of studies. While the NOS is a widely recognized tool for assessing the risk of bias, it may not fully capture the complexities associated with PTG. PTG involves multidimensional psychological, social, and emotional processes, which may be influenced by a variety of factors not entirely accounted for by the NOS. As such, relying solely on the NOS may limit the ability to assess the nuanced methodological challenges present in studies of PTG. Future research may benefit from supplementing the NOS with other bias assessment tools or qualitative methodologies to provide a more comprehensive assessment of study quality and the contextual factors influencing PTG outcomes. This paper will be submitted for peer-reviewed publication. It will also be part of a thesis submitted to the University of Strathclyde.

## Appendix

Multimedia Appendix 1 Search strategy.

Multimedia Appendix 2 Systematic review and meta-analysis data extraction form.

Multimedia Appendix 3 PRISMA-P (Preferred Reporting Items for Systematic review and Meta-Analysis Protocols) 2015 checklist.

## Abbreviations

MeSH: Medical Subject Headings
NOS: Newcastle-Ottawa Scale
PRISMA: Preferred Reporting Items for Systematic Reviews and Meta-Analyses
PRISMA-P: Preferred Reporting Items for Systematic Reviews and Meta-Analyses Protocols
PTG: posttraumatic growth
PTGI: Posttraumatic Growth Inventory
PTSD: posttraumatic stress disorder

## Data Availability

All data generated or analyzed during this study are included in this published article and Multimedia Appendices 1-3.

## References

[1] Levi-Belz Y, Hamdan S (2023). Shame, depression, and complicated grief among suicide loss-survivors: the moderating role of self-disclosure. Eur J Psychotraumatol.

[2] Pitman A, Osborn D, King M, Erlangsen A (2014). Effects of suicide bereavement on mental health and suicide risk. Lancet Psychiatry.

[3] (2021). Suicide worldwide in 2019: global health estimates. World Health Organization.

[4] Andriessen K, Rahman B, Draper B, Dudley M, Mitchell PB (2017). Prevalence of exposure to suicide: a meta-analysis of population-based studies. J Psychiatr Res.

[5] Cerel J, Brown MM, Maple M, Singleton M, van de Venne J, Moore M, Flaherty C (2019). How many people are exposed to suicide? not six. Suicide Life Threat Behav.

[6] Andriessen K, Krysinska K, Grad OT (2017). Postvention in Action: The International Handbook of Suicide Bereavement Support.

[7] Grad O, O'Connor R, Platt S, Gordon J (2011). The sequelae of suicide: survivors. International Handbook of Suicide Prevention: Research, Policy and Practice.

[8] Mitchell A, Terhorst L (2017). PTSD symptoms in survivors bereaved by the suicide of a significant other. J Am Psychiatr Nurses Assoc.

[9] Tal Young I, Iglewicz A, Glorioso D, Lanouette N, Seay K, Ilapakurti M, Zisook S (2012). Suicide bereavement and complicated grief. Dialogues Clin Neurosci.

[10] Tedeschi R, Calhoun LG (2004). Target Article: "Posttraumatic Growth: Conceptual Foundations and Empirical Evidence". Psychological Inquiry.

[11] Wu X, Kaminga AC, Dai W, Deng J, Wang Z, Pan X, Liu A (2019). The prevalence of moderate-to-high posttraumatic growth: a systematic review and meta-analysis. J Affect Disord.

[12] Mattson E, James L, Engdahl B (2018). Personality factors and their impact on PTSD and post-traumatic growth is mediated by coping style among OIF/OEF veterans. Mil Med.

[13] Jian Y, Hu T, Zong Y, Tang W (2022). Relationship between post-traumatic disorder and posttraumatic growth in COVID-19 home-confined adolescents: the moderating role of self-efficacy. Curr Psychol.

[14] Tedeschi RG, Calhoun LG (1996). The posttraumatic growth inventory: measuring the positive legacy of trauma. J Trauma Stress.

[15] Feigelman W, Jordan JR, Gorman BS (2009). Personal growth after a suicide loss: cross-sectional findings suggest growth after loss may be associated with better mental health among survivors. Omega (Westport).

[16] Park CL, Cohen LH, Murch RL (1996). Assessment and prediction of stress-related growth. J Pers.

[17] Frazier P, Conlon A, Glaser T (2001). Positive and negative life changes following sexual assault. J Consult Clin Psychol.

[18] Pitman A, De Souza T, Khrisna Putri A, Stevenson F, King M, Osborn D, Morant N (2018). Support needs and experiences of people bereaved by suicide: qualitative findings from a cross-sectional british study of bereaved young adults. Int J Environ Res Public Health.

[19] Chapple A, Ziebland S, Hawton K (2015). Taboo and the different death? Perceptions of those bereaved by suicide or other traumatic death. Sociol Health Illn.

[20] Barnes D (2006). The aftermath of suicide among African Americans. Journal of Black Psychology.

[21] Peters K, Cunningham C, Murphy G, Jackson D (2016). Helpful and unhelpful responses after suicide: Experiences of bereaved family members. Int J Ment Health Nurs.

[22] Pitman A, Rantell K, Moran P, Sireling L, Marston L, King M, Osborn D (2017). Support received after bereavement by suicide and other sudden deaths: a cross-sectional UK study of 3432 young bereaved adults. BMJ Open.

[23] Levi-Belz Y, Krysinska K, Andriessen K (2021). "Turning personal tragedy into triumph": a systematic review and meta-analysis of studies on posttraumatic growth among suicide-loss survivors. Psychol Trauma.

[24] Moher D, Tsertsvadze A, Tricco AC, Eccles M, Grimshaw J, Sampson M, Barrowman N (2008). When and how to update systematic reviews. Cochrane Database Syst Rev.

[25] Gopalakrishnan S, Ganeshkumar P (2013). Systematic reviews and meta-analysis: understanding the best evidence in primary healthcare. J Family Med Prim Care.

[26] Moher D, Liberati A, Tetzlaff J, Altman DG (2009). Preferred reporting items for systematic reviews and meta-analyses: the PRISMA statement. PLoS Med.

[27] Shamseer L, Moher D, Clarke M, Ghersi D, Liberati A, Petticrew M, Shekelle P, Stewart LA, PRISMA-P Group (2015). Preferred reporting items for systematic review and meta-analysis protocols (PRISMA-P) 2015: elaboration and explanation. BMJ.

[28] Covidence Systematic Review Software.

[29] Wells GA, Shea B, O'Connell D, Peterson J, Welch V, Losos M, Tugwell P (2013). The Newcastle-Ottawa Scale (NOS) for assessing the quality of nonrandomised studies in meta-analyses.

[30] The R Project for Statistical Computing. R Foundation for Statistical Computing.

[31] Ben-Shachar MS, Lüdecke D, Makowski D (2020). effectsize: estimation of effect size indices and standardized parameters. JOSS.

[32] Lüdecke D (2019). escffect Size Computation for Meta Analysis (Version 0. Zenodo.

[33] Viechtbauer W (2010). Conducting meta-analyses in R with the metafor package. J. Stat. Soft.

[34] Hedges LV, Vevea JL (1998). Fixed- and random-effects models in meta-analysis. Psychological Methods.

[35] Borenstein M (2024). Avoiding common mistakes in meta-analysis: Understanding the distinct roles of Q, I-squared, tau-squared, and the prediction interval in reporting heterogeneity. Res Synth Methods.

[36] Peters JL, Sutton AJ, Jones DR, Abrams KR, Rushton L (2008). Contour-enhanced meta-analysis funnel plots help distinguish publication bias from other causes of asymmetry. J Clin Epidemiol.

[37] Harrer M, Cuijpers P, Furukawa T, Ebert DD (2012). dmetar: Companion R Package For The Guide 'Doing Meta-Analysis in R'. R package version 0.1.0.

