# Supplementary figures and images for: Posttraumatic Growth Among Suicide-Loss Survivors: Protocol for an Updated Systematic Review and Meta-Analysis
Source: JMIR Res Protoc. 2025 Feb 14;14:e64615. doi: 10.2196/64615 (PMC11888060; doi:10.2196/64615)

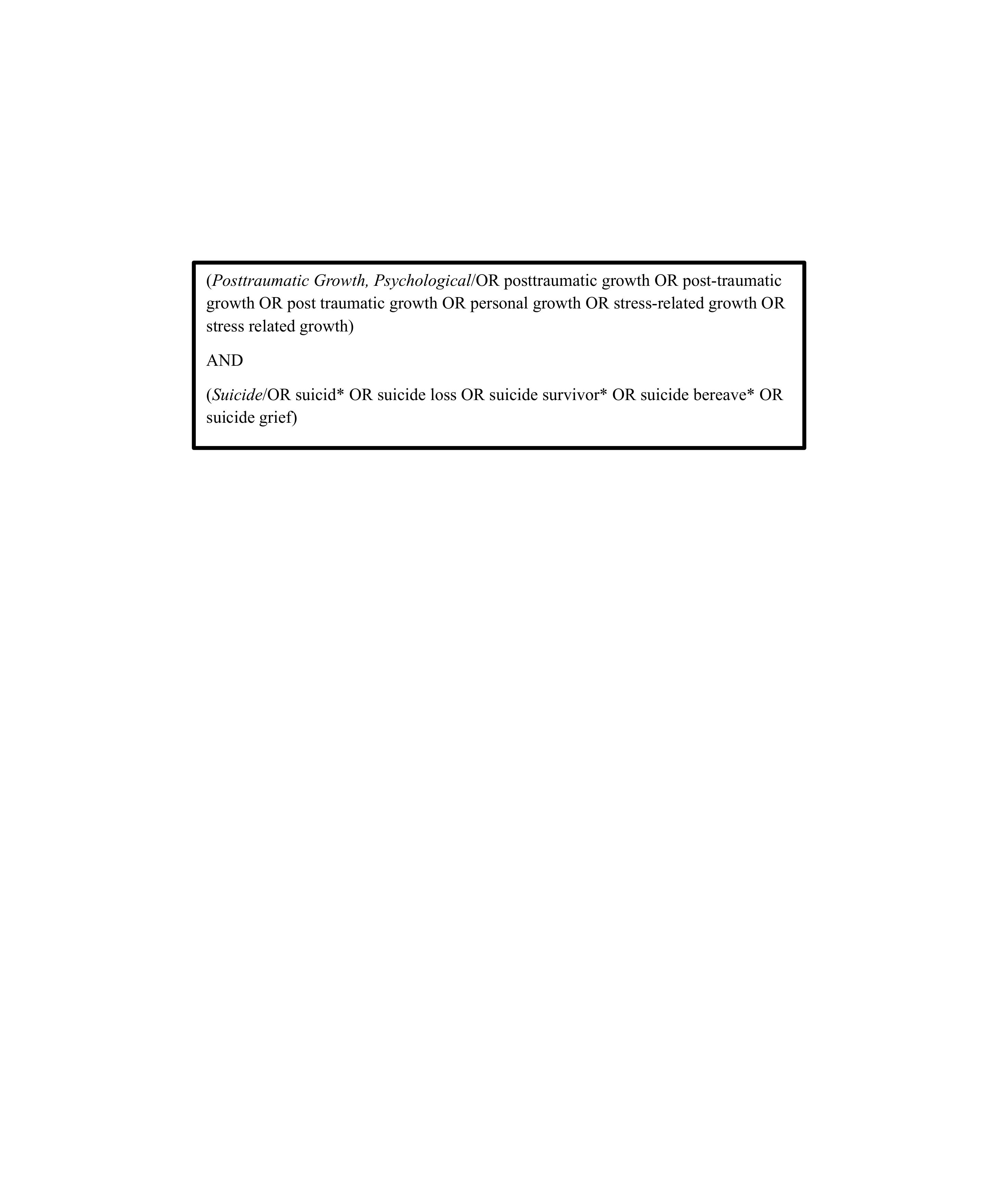

Supplement: Multimedia Appendix 1 [file resprot_v14i1e64615_app1.png]
